# A89 PERCEPTIONS OF NON-TECHNICAL SKILLS IN GASTROINTESTINAL ENDOSCOPY: A THEMATIC ANALYSIS OF FOUR FOCUS GROUPS

**DOI:** 10.1093/jcag/gwab049.088

**Published:** 2022-02-21

**Authors:** M Scaffidi, N Gimpaya, C Pattni, S Genis, R Khan, J Li, R Bansal, S Grover

**Affiliations:** St. Michael’s Hospital, Toronto, ON, Canada

## Abstract

**Background:**

Nontechnical skills (NTS), which involve an individual’s cognitive, attitudinal, and social skills that supplement task expertise, are an essential component in the practice of gastrointestinal endoscopy. There is a growing body of literature that highlights the association between these skills and patient outcomes. To date, however, these skills have not been adequately defined within the context of gastrointestinal endoscopy.

**Aims:**

To define the domain and corresponding characteristics of NTS in GI endoscopy.

**Methods:**

We conducted a qualitative study at a tertiary-care academic center in Toronto, Ontario. Specifically, we held four focus groups with physician endoscopists, nurses who work in an endoscopy unit, and patients who have had previous endoscopies, in order to ascertain their input on the role of NTS in gastrointestinal endoscopy. The three groups were interviewed independently and there was one focus group of both physicians and nurses that was used for validation of our initial thematic framework. Data from the focus groups was collected using a combination of field notes and discussion transcriptions. Three authors independently generated codes from these data. Using these codes, a thematic network analysis was used to identify emerging themes. The primary outcome of this study was the development of a cohesive thematic network of NTS in endoscopy, including their characteristics and examples.

**Results:**

The four focus groups included a total of 34 participants, including 15 physician endoscopists, 15 nurses, and 4 patients. Using thematic network analysis, we identified six dimensions of NTS using the first three focus groups: communication; professionalism; teamwork; leadership; decision-making; and situational awareness. Additional topics related to the practice and evaluation of NTS were identified. In particular, there is a degree of subjectivity in the appraisal of NTS due to the nuances among individual practice, aside from egregious errors of NTS (e.g. unprofessional behaviours). The use of video recordings was suggested as a way to capture signs of good NTS, such as appropriate levels of calmness during procedures and attention to patient comfort. Finally, patient involvement can be useful for evaluating communication and professionalism based on patient comprehension and the nature of the therapeutic relationship.

**Conclusions:**

Our findings provide the first cohesive framework of NTS in gastrointestinal endoscopy that is anchored in real world experiences with relevant stakeholders – physicians, nurses, and patients. Future research should consolidate these findings into an assessment tool for NTS in order to evaluate and provide feedback to endoscopists who are both in training and in practice.

**Funding Agencies:**

CAG

